# Supplementary material for: Causal Relationship between Aging and Anorexia Nervosa: A White-Matter-Microstructure-Mediated Mendelian Randomization Analysis
Source: Biomedicines. 2024 Aug 16;12(8):1874. doi: 10.3390/biomedicines12081874 (PMC11351342; doi:10.3390/biomedicines12081874)
Supplement: Supplementary file 1 [file biomedicines-12-01874-s001.zip › Supplementary Figure/Figure S4/Singe SNP plot of MR analysis for TL to ubm-b-1729.pdf]

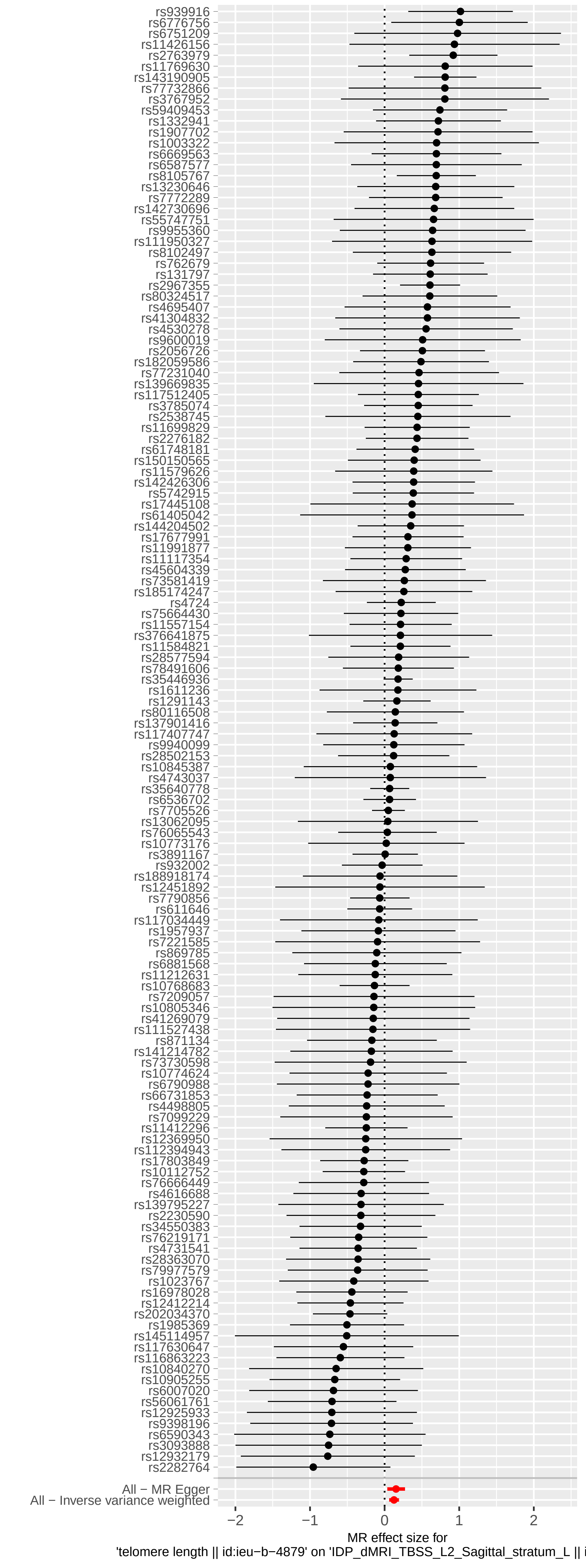
rs939916
rs6776756
rs6751209
rs11426156
rs2763979
rs11769630
rs143190905
rs77732866
rs3767952
rs59409453
rs1332941
rs1907702
rs1003322
rs6669563
rs6587577
rs8105767
rs13230646
rs7772289
rs142730696
rs55747751
rs9955360
rs111950327
rs8102497
rs762679
rs131797
rs2967355
rs80324517
rs4695407
rs41304832
rs4530278
rs9600019
rs2056726
rs182059586
rs77231040
rs139669835
rs117512405
rs3785074
rs2538745
rs11699829
rs2276182
rs61748181
rs150150565
rs11579626
rs142426306
rs5742915
rs17445108
rs61405042
rs144204502
rs17677991
rs11991877
rs11117354
rs45604339
rs73581419
rs185174247
rs4724
rs75664430
rs11557154
rs376641875
rs11584821
rs28577594
rs78491606
rs35446936
rs1611236
rs1291143
rs80116508
rs137901416
rs117407747
rs9940099
rs28502153
rs10845387
rs4743037
rs35640778
rs6536702
rs7705526
rs13062095
rs76065543
rs10773176
rs3891167
rs932002
rs188918174
rs12451892
rs7790856
rs611646
rs117034449
rs1957937
rs7221585
rs869785
rs6881568
rs11212631
rs10768683
rs7209057
rs10805346
rs41269079
rs111527438
rs871134
rs141214782
rs73730598
rs10774624
rs6790988
rs66731853
rs4498805
rs7099229
rs11412296
rs12369950
rs112394943
rs17803849
rs10112752
rs76666449
rs4616688
rs139795227
rs2230590
rs34550383
rs76219171
rs4731541
rs28363070
rs79977579
rs1023767
rs16978028
rs12412214
rs202034370
rs1985369
rs145114957
rs117630647
rs116863223
rs10840270
rs10905255
rs6007020
rs56061761
rs12925933
rs9398196
rs6590343
rs3093888
rs12932179
rs2282764
All − MR Egger
All − Inverse variance weighted
−2
−1
0
1
2
MR effect size for
'telomere length || id:ieu−b−4879' on 'IDP_dMRI_TBSS_L2_Sagittal_stratum_L || i
